# Increased energy use for adaptation significantly impacts mitigation pathways

**DOI:** 10.1038/s41467-022-32471-1

**Published:** 2022-08-24

**Authors:** Francesco Pietro Colelli, Johannes Emmerling, Giacomo Marangoni, Malcolm N. Mistry, Enrica De Cian

**Affiliations:** 1 Department of Economics, Ca’ Foscari University of Venice, 30121 Venice, Italy; 2 Fondazione Centro Euro-Mediterraneo sui Cambiamenti Climatici (CMCC), 30175 Venice, Italy; 3 RFF-CMCC European Institute on Economics and the Environment (EIEE), Fondazione Centro Euro-Mediterraneo sui Cambiamenti Climatici, 20144 Milan, Italy; 4 Department of Economics, Management and Industrial Engineering, Politecnico di Milano, 20156 Milan, Italy; 5 Department of Public Health, Environments and Society, London School of Hygiene & Tropical Medicine, London, WC1H 9SH UK

**Keywords:** Climate-change policy, Energy and society, Climate-change adaptation

## Abstract

Climate adaptation actions can be energy-intensive, but how adaptation feeds back into the energy system and the environment is absent in nearly all up-to-date energy scenarios. Here we quantify the impacts of adaptation actions entailing direct changes in final energy use on energy investments and costs, greenhouse gas emissions, and air pollution. We find that energy needs for adaptation increase considerably over time and with warming. The resulting addition in capacity for power generation leads to higher greenhouse gas emissions, local air pollutants, and energy system costs. In the short to medium term, much of the added capacity for power generation is fossil-fuel based. We show that mitigation pathways accounting for the adaptation-energy feedback would require a higher global carbon price, between 5% and 30% higher. Because of the benefits in terms of reduced adaptation needs, energy system costs in ambitious mitigation scenarios would be lower than previous estimates, and they would turn negative in well-below-2-degree scenarios, pointing at net gains in terms of power system costs.

## Abstract (web summary)

A new study characterizes adaptation in mitigation pathways, and shows that climate adaptation can lead to higher energy demand, power system costs and carbon prices, with mitigation’s benefits compensating decarbonization costs.

## Introduction

In its latest assessment, the Intergovernmental Panel on Climate Change (IPCC) reports with high confidence that adaptation actions focusing on sectorial and short-term benefits can lead to maladaptive responses and build up risk over time^1^. The Illustrative Mitigation Pathways (IMPs) developed by Working Group III do not account for adaptation costs, and we still lack a comprehensive characterization of mitigation pathways in the presence of adaptation actions. When people adjust to experienced or expected changes in climate, their actions often involve modifications of energy expenditure or the purchase of new and often more efficient appliances^2^. Many of the adaptation actions that individuals and industries have implemented so far are energy-intensive^3,4^. Some examples include water pumping, desalinization, and water purification^5,6^. Growing evidence shows that higher temperatures and more frequent and prolonged extremes lead to more electricity for space cooling^7–9^, for refrigeration^10^, and for entertainment appliances if people spend more time indoors^11^. While space heating is expected to require less energy^12–18^, the extent and occurrence of cold waves can actually go in the opposite direction^19^. Extreme temperatures also directly affect labor and capital productivity^20^, leading industrial and commercial activities to adjust their energy usage as well. The impacts of heat on labor productivity are well-documented^21^, and air-conditioning can reduce production losses in the manufacturing and service sectors^22^. The performance of equipment, such as data centers, and the mechanical functioning of machines are also sensitive to the surrounding temperature conditions, and high operating temperatures can cause electronic components to lose functionality^23^.

These are examples of adaptation actions that would have direct impacts on the energy system, with ultimate feedback on the climate and the environment. Since low-energy-demand development pathways increase the flexibility needed to achieve low-temperature mitigation scenarios and reduce the need for negative emissions^24^, these actions jeopardize achieving low-carbon targets^25^. The sensitivity of energy demand to weather fluctuations has long been documented in economic and engineering studies^26–29^. Yet, most energy scenarios and mitigation pathways do not include the adaptation-energy feedback^30^, and only very few studies have used IAMs to conduct macroeconomic assessments at the global scale^4^. Global-scale contributions have relied on econometric simulations (see refs. 17, 31, 32) to provide partial equilibrium projections of the potential, ex-ante changes in energy demand without accounting for price-induced substitution and income effects that only macroeconomic approaches can describe. Although Computable General Equilibrium (CGE) models suggest that the global market economy can easily absorb the costs associated with changes in energy use for adaptation^33–36^, we lack an overall understanding of the implications for the energy system in the context of ambitious mitigation policies.

Here we provide evidence on the macroeconomic implications of climate change impacts and analyze how price-induced substitution and income effects, as well as technical change adjustments, affect global and regional mitigation pathways. We integrate an adaptation-energy feedback loop for all world regions, main fuels, and economic sectors into the IAM "World Induced Technical Change Hybrid model" (WITCH)^37^. Our results indicate that adapting to climate change by means of the energy habits as we did in the past will increase the global demand for electricity by 7% (18%) and for fuels by 1% (2.5%) by 2050 (2100) under the current socioeconomic trends and mitigation policies. The increase in energy needs leads to more physical capital being locked into fossil fuels, for an additional 960 Gigawatt (GW) of new gas-fired capacity, 360 GW of new oil-fired capacity, and 300 GW of new coal-fired capacity, cumulatively from 2020 to 2050 (corresponding to a yearly average increase in new fossil-fuel-based capacity of 55 GW). Adaptation would also require more resources for grid investments, power generation, and, in some regions and sectors, fuel consumption. The carbon price required to reach a certain carbon budget would need to increase, and the cost-effective allocation of emissions would also look different compared to a situation that does not account for the energy use for adaptation. We find that when the energy requirements of adaptation are modeled, the gains from lower adaptation needs reduce the additional energy system costs associated with more ambitious mitigation goals. Our study endogenously integrates the energy needs for adaptation into mitigation pathways, highlighting the implications for decarbonization and policy design.

## Results

### An integrated assessment of the energy needs for adaptation

IAMs couple human and climate systems and quantitatively describe the interdependencies among socioeconomic, behavioral, technological, and physical drivers affecting future global and regional pathways. The WITCH model^37^ is a process-detailed IAM that fully integrates into the optimization process a top-down representation of the economy, a bottom-up description of the energy system, simplified dynamics of the climate system, and an air pollution module (See Methods).

**Fig. 1:**
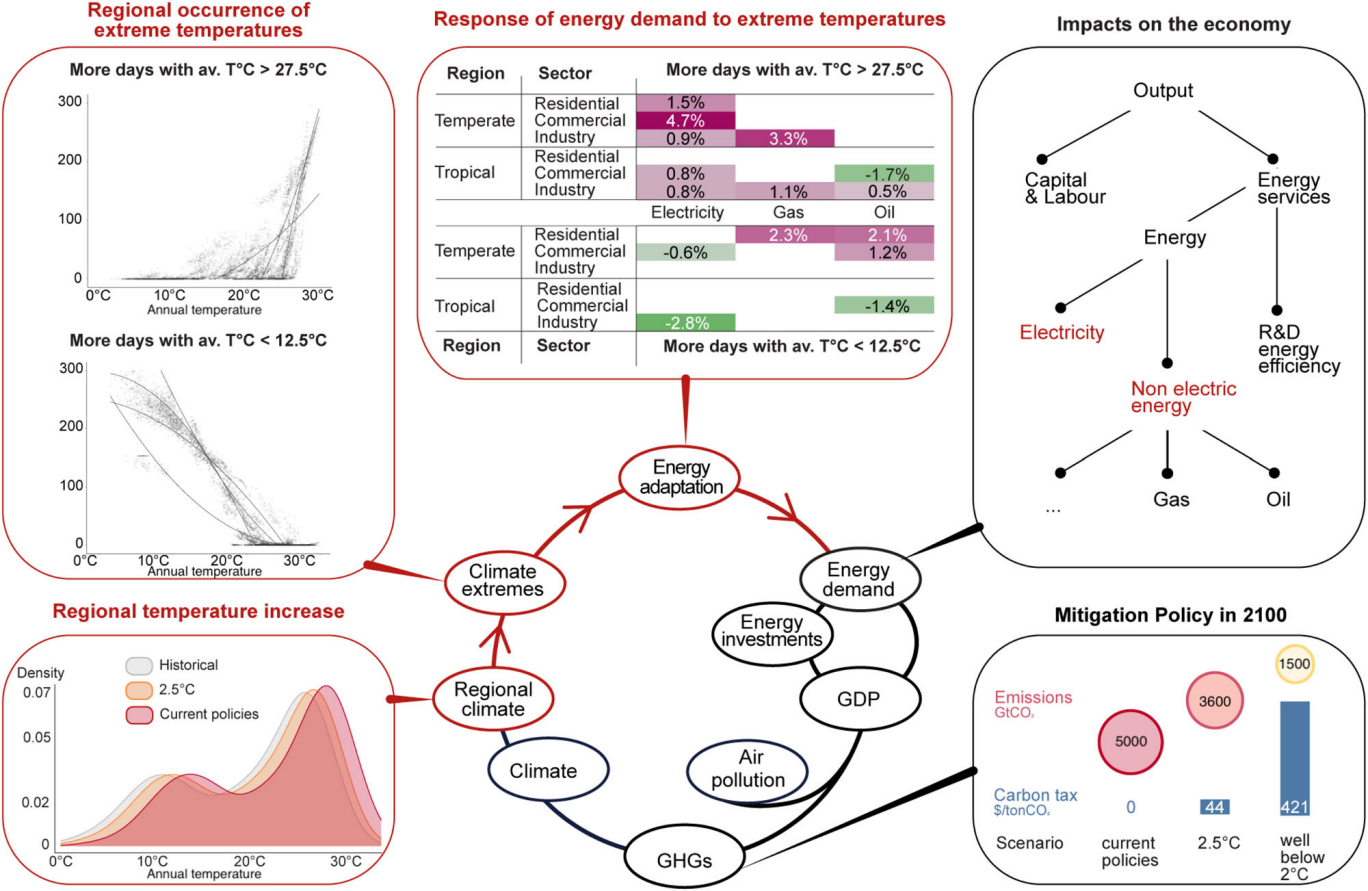
Integrated approach to the adaptation-energy feedback loop. The circle represents the integrated framework of the World Induced Technical Change Hybrid (WITCH) model, linking the economy, the energy system, and the climate system. Red lines indicate the components modified with new equations to model the adaptation-energy feedback loop. Top-left panel: eight clusters characterize the heterogeneity in the relationship between the Extreme Temperature Indicators (ETIs) and annual average temperature across world regions. Top-central panel: semi-elasticities as estimated in ref. 31 representing the percentage change in energy demand for one additional day with average daily temperature (T) in the upper (T > 27. 5 °C)/lower (T < 12. 5 °C) bin, see ref. 31. A detailed description of each step and of the methodological advancements is presented in the Methods and Supplementary Methods. The WITCH model version used for the analysis (WITCH 5.0) is described in detail in ref. 37.

We model the adaptation-energy feedback loop in three steps summarized in Fig. 1. First, we empirically estimate a reduced-form relationship between country-level annual average temperature and two extreme temperature indicators (ETIs), the annual occurrence of extreme cold (<12.5 °C) and hot (>27.5 °C) days (Supplementary Methods). Cluster analysis is used to capture the heterogeneity in the reduced-form equation across countries with markedly different climates (four clusters shown in the top-left panel of Fig. 1). The resulting statistical emulator makes it possible to directly project the future occurrence of days with extreme temperatures based on the regional annual temperature levels. Regional temperatures are also statistically related to the global change in annual mean temperature, the variable commonly included in the climate modules of IAMs (See Methods and Supplementary Methods).

Second, we model the relationship between changes in the occurrence of extreme temperature days (ETIs) and the demand for electricity, gas, and oil in the residential, commercial, and industrial sectors after the empirical estimates provided in ref. 31, (see Methods, Supplementary Methods and Supplementary Fig. 2). This approach differs from analyses based on indicators of Cooling and Heating Degree Days (CDDs and HDDs) or average annual temperatures (see refs. 13, 14, 36, 38) that tend to shrink the tails of the distribution of meteorological drivers, leading to an aggregation bias that can underestimation of the impacts on energy demand. A comparison between the two approaches is provided by ref. 39, who shows how modeling energy consumption with HDDs and CDDs does not make it possible to capture the nonlinear increase in energy consumption at extremely high temperatures.

Third, changes in energy demand affect the economy, described by the model’s production tree, through the productivity of energy inputs, which is now endogenous. The supply-side of the energy sector endogenously adjusts to meet the climate-induced changes in demand, leading to changes in the costs of power generation, grid infrastructure, fuel extraction, and expenditures, including domestic extraction and imports.

We examine the implications of the adaptation–energy feedback on mitigation policies (carbon pricing and cost-effective emission allocation) and their co-benefits in terms of air pollution in a cost-effective setting. The carbon budget is consistent with a predetermined climate target and implemented via a uniform global carbon price. We focus on climate policies that achieve the goal of keeping global average temperature increases either around 2.5 °C or well-below 2 °C compared to the preindustrial level (see Supplementary Methods). Climate targets are therefore achieved in a cost-optimal way, with neither international compensations nor carbon emission trading. In the current policy scenario, countries maintain the implemented climate policies until 2020 and a similar level of climate ambition is assumed afterwards. Socioeconomic trends of population and output growth follow the middle-of-the-road Shared Socioeconomic Pathway SSP2^40^, while results for other SSPs are presented as sensitivity analysis.

### Regional exposure to extreme temperatures

**Fig. 2:**
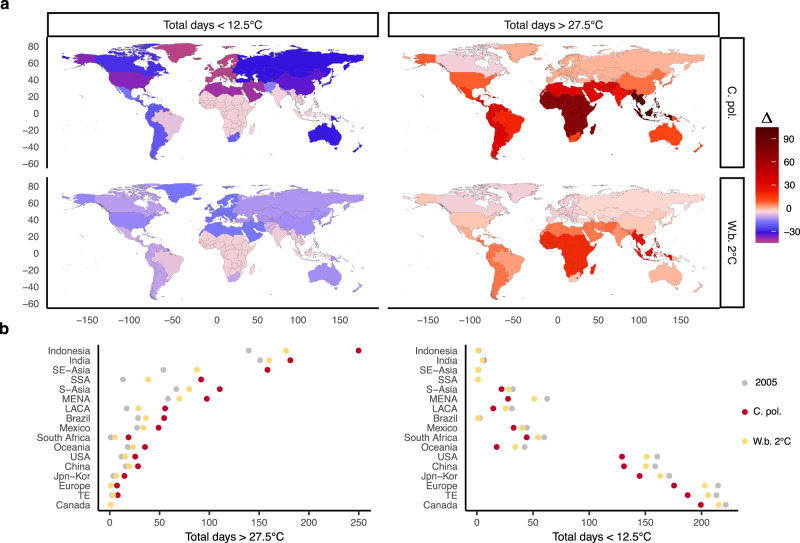
Future changes in the frequency of warm and cold days. **a** Difference (Δ) between future (2090–2100) and historical (2005) annual number of days with average daily temperature (T) > 27.5 °C and T < 12. 5 °C. **b** Regional count of total days with T > 27. 5 °C and T < 12. 5 °C in 2005 and in 2100 by policy scenario. Temperature indicators are constructed with population-weighted daily temperatures. Scenarios: Current policies (C.Pol) and well-below 2 °C (W.b. 2 °C).

Under the current policy scenario, the annual count of warm days (>27.5 °C) at around 2100 goes up substantially at the global level. The increase in the annual number of warm days, compared to the historical level, exceeds the decrease in the annual number of cold days (<12.5 °C). Maps of future projections point to a large variation in regional exposure (Fig. 2). The populations in Indonesia, South-East Asia and Sub-Saharan Africa are projected to experience, by the end of the century, more than 100 additional days with average temperatures above 27.5 °C, with respect to the simulated exposure in the year 2005. The implications for temperate economies are also non-negligible: at around 2100, in the current policy scenario, the United States and China are projected to experience an annual number of warm days that match the historical level experienced in Mexico. Europe, the Middle East, and the United States experience the largest decrease in the number of cold days. Stringent mitigation policies drastically reduce the exposure to extreme warm days and, in the well below 2 °C scenarios, the projected median number of additional days above 27.5 °C at around 2100 is about three times smaller compared to the current policy scenario.

### Final energy demand for adaptation

**Fig. 3:**
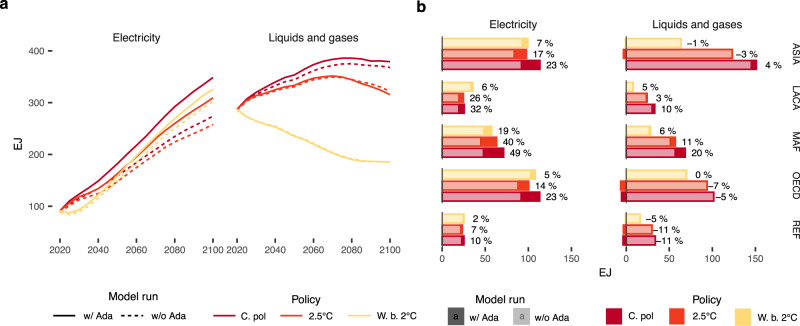
Projected electricity and fuel (including liquids and gases) demand for adaptation under Shared Socioeconomic Pathway (SSP) 2 assumptions. **a** Annual global average demand from 2020 to 2100 across the different scenarios excluding (dotted) and including (solid) the adaptation-energy feedback under the SSP2. **b** Regional final energy demand in 2100. Light bars show the value excluding the adaptation-energy feedback, while dark stacked bars show the positive or negative variation in energy demand induced by the adaptation-energy feedback. Labels in panel b show the regional percentage increase. Scenarios: Current policies (C.Pol), 2. 5 °C and well-below 2 °C (W.b. 2 °C).

Energy needs for adaptation increase over time and with the degree of global warming (Fig. 3a). Adaptation-energy demand in buildings and industry rise considerably in the current policy scenario. Global electricity will increase by 18% (an additional 75 EJ) in 2100, compared to the projected demand in the same year but without adaptation. The final demand for liquids and gases increases by 2.5% (an additional 10 EJ in 2100). Table 1 presents the total and relative increase in the combined final energy demand for electricity and fuels due to adaptation across policy scenarios and SSPs. The overall amount of energy required for adaptation in 2100 under the SSP2, current policy scenario is equal to 20% of the global final energy demand in 2019^41^. Different assumptions on the baseline energy demand, as implied by different socioeconomic pathways, affect the quantification of the additional energy use for adaptation, which reaches over 100 EJ / year in 2100 in the SSP5 (see Table 1 and Supplementary Results).

**Table 1:** Global final energy demand (EJ/year) in 2100 by scenario with (w Ada) and without adaptation (w/o Ada)

	Current policy		2.5 °C		Well-below 2 °C	
	w/o Ada	w Ada	w/o Ada	w Ada	w/o Ada	w Ada
SSP2	641	727 (+13%)	579	624 (+8%)	486	510 (+5%)
SSP3	545	608 (+12%)	503	542 (+8%)	411	436 (+6%)
SSP5	771	889 (+15%)	688	748 (+9%)	610	647 (+6%)

Ambitious mitigation policies cut the energy use for adaptation by half in the moderate emissions scenario (2.5 °C) and by more than 70% in the low emissions scenario (well-below 2 °C). The demand for liquids and gases for adaptation would essentially reduce to zero. We find that the majority of the additional energy needs are met by using electricity in both residential and commercial buildings and industrial activities. The industrial sector accounts for 40% of the additional electricity requirements (see Supplementary Fig. 4). Heating, ventilation, and air-conditioning (HVAC) systems used by industries include comfort-related energy use and continuous or process-related HVAC, the latter ensuring that the operation of manufacturing systems and production processes (e.g., food processing and storage industry) is not undermined by temperature variations^42^.

The small increase in the final demand for liquids and gases masks heterogeneous responses across sectors. The reduction in fuel demand from lower heating requirements in residential and commercial buildings is compensated by the increase in industrial fuel demand as a response to more hot days. While space cooling in residential buildings is mostly delivered through electricity, industrial and commercial facilities can use fossil-fueled based cooling techniques, such as cooling absorption^43^. Variations in the consumption of fuels for cooling and heating purposes can also result from fuel-switching practices. For instance, the use of distributed petroleum-fired generators to satisfy final electricity demand may be particularly relevant in developing tropical economies characterized by unreliable electricity distribution systems.

Africa and the Middle East (MAF) will face the largest relative increase in final energy demand for adaptation (Fig. 3b). These two regions account for roughly one-fourth of the global additional increase in electricity demand, rising by almost 50% in the current policy scenario in 2100 relative to the no-adaptation case. These results indicate that the largest relative increase in electricity demand occurs in places with power systems poorly prepared to face peaks in power demand for cooling^44^. If these energy requirements cannot be met, extreme temperatures can create health emergencies in developing countries, and this could be one additional channel through which ineffective adaptation may further reinforce global inequalities.

### New power capacity requirements

**Fig. 4:**
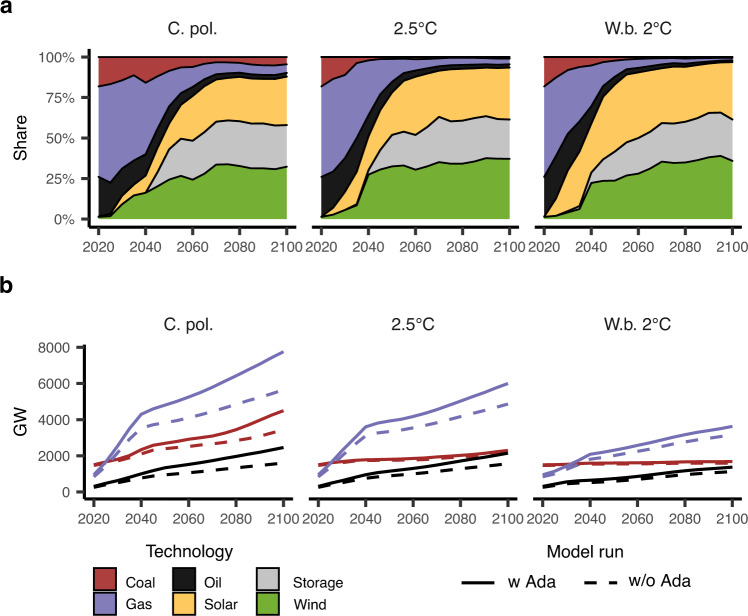
Additional power generation capacity. **a** Technology mix of the additional average annual capacity to fulfill the additional energy for adaptation. **b** Additional fossil-based new capacity installed cumulatively with (solid lines) and without (dotted lines) the adaptation-energy feedback. The additional new capacity installed cumulatively including also renewable sources is presented in Supplementary Fig. 6. The technologies unaffected by the adaptation-energy feedback are not included. Scenarios: Current policies (C.Pol), 2.5 °C and well-below 2 °C (W.b. 2 °C).

Additional new generation capacity is required to accommodate the increase in electricity use for adaptation. The mix of the additional generation capacity will be shaped by the ambitiousness and timing of mitigation policies (Fig. 4a). In the next three decades (2020–2050), capacity additions in the current policy scenario will be still carbon-intensive, as mitigation policies start to redirect power investments progressively over time. After 2050, the new capacity mostly consists of renewable energy and storage.

Climate policy is key to avoid negative feedback on the energy use for adaptation to mitigation objectives. If climate policy is not ambitious enough, adaptation needs can lead to additional lock-in into fossil-based generation (Fig. 4b and Supplementary Fig. 6): in the current policy scenario, an additional 300 GW of new coal-fired capacity, 390 GW of new oil-fired capacity, and 960 GW of new gas-fired capacity are installed cumulatively by 2050, as a result of the adaptation feedback, an average yearly addition of 55 GW for the three technologies combined. The additional oil-fired and coal-fired capacity required by the adaptation-energy feedback by 2050 falls by 50–90% from the current policy scenario, depending on the stringency of the climate policy. Additional gas-fired generation falls more progressively, and still, 300–580 GW new capacity is installed to meet adaptation needs cumulatively by 2050, in the ambitious policy scenarios. Reduction in the additional investments in fossil-fuel capacity in the climate policy scenarios results from the combination of lower electricity demand increases due to milder climate change as well as from the variation in the cost-optimal generation mix.

The share of fossil-based generation in the total power mix does not change considerably when energy for adaptation is accounted for (see Table 2). While we find non-negligible changes in the total carbon intensity of power generation in the current policy scenario, the overall total carbon intensity of the energy system does not change considerably in any scenario (see Table 2 and Supplementary Fig. 5). The energy use for adaptation poses new challenges to the mitigation goals mostly through the shift in demand, which increases in the energy intensity of the economy.

**Table 2:** Impact of energy use for adaptation on the power generation mix with (w Ada) and without adaptation (w/o Ada)

	Current policy		2.5 °C		Well-below 2 °C	
	w/o Ada	w Ada	w/o Ada	w Ada	w/o Ada	w Ada
**Share of fossil fuels in the power generation mix**						
2030	47%	49%	45%	47%	37%	38%
2050	23%	25%	17%	18%	12%	12%
2100	6%	7%	4%	4%	2%	2%
**Carbon intensity of power generation (gCO**_**2**_**/kWh)**						
2030	460	471	434	441	185	199
2050	306	325	171	169	≈0	≈0
2100	118	144	44	30	−121	−113

### Variation in the energy system costs

**Fig. 5:**
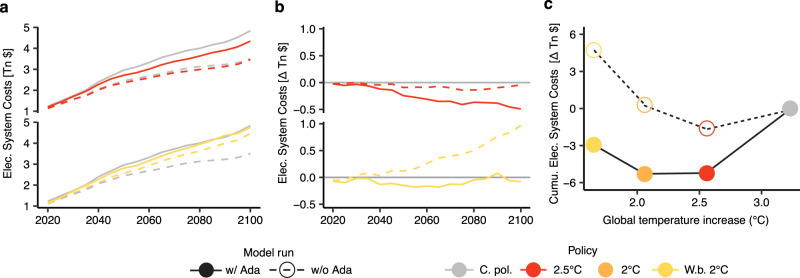
Annual electricity system costs by scenario. **a** Total electricity system costs in trillion $, 2005 Purchasing Power Parity (PPP). **b** Additional electricity system costs in the mitigation scenarios with respect to the current policy, in trillion $(2005, PPP). **c** Variation in the cumulative electricity system costs associated to the more ambitious mitigation policy scenarios with respect to the current policy, in trillion $(2005, PPP). All projections are presented alternatively for the case with (solid lines) or without (dotted lines) adaptation. Operative fuel expenses for fossil-based power generation are included in the electricity system costs. Scenarios: Current policies (C.Pol), 2.5 °C and well-below 2 °C (W.b. 2 °C). Results presented in panel **a** and **b** for the scenario 2 °C are not shown to avoid clutter and can be fund in the Supplementary Material.

The supply-side adjustments needed to meet additional energy for adaptation have non-negligible economic implications. The energy-adaptation feedback increases supply-side energy system costs (ESC), combining power and fuels costs, in all policy scenarios (Fig. 5a). The increase is mostly driven by power system costs, including new investments in generation capacity, grid investments, and operating expenses from fuel consumption of traditional power plants (Supplementary Fig. 7). In the current policy scenario, global costs for electricity supply rise by 21% (Net Present Value incurred from 2020 to 2100), while total ESC increase by 4.5% (Table 3), due to both higher final energy demand and higher energy prices. The additional supply-side costs are passed on to consumers through increases in the price of electricity, growing by 2–6% due to the adaptation-energy feedback, depending on the year, scenario, and region (see Supplementary Fig. 8).

**Table 3:** Energy System Costs (ESC) in Net Present Value ($ NPV, 3% discount rate) by policy scenario with (w/ Ada) and without adaptation (w/o Ada)

	Current policy		2.5 °C		Well-below 2 °C	
	w/o Ada	w/ Ada	w/o Ada	w/ Ada	w/o Ada	w/ Ada
Electricity	66	80	65	74	71	77
Change (%)	—	—	−1 (−2%)	−5 (−6%)	5 (7%)	−3 (−4%)
Liquids and gases	297	299	291	290	271	271
Change (%)	—	—	−6 (−2%)	−9 (–3%)	−26 (−9%)	−28 (−9%)

Ambitious mitigation scenarios can cut the increase in the ESC induced by adaptation by more than half, depending on the stringency of the climate target. Most importantly, we find that when the adaptation feedback is included, the gains from lower adaptation needs reduce considerably the additional power system costs required to reach ambitious mitigation targets (Fig. 5b, c and Table 3). Even ambitious mitigation ("Well-below 2 °C" scenario) can entail net gains in terms of power system costs compared to the current policy scenario. Our results underscore that ignoring the energy system costs attributable to rising energy use for adaptation results in an overestimation of the additional costs of mitigation policies (for the results across SSPs see Supplementary Fig. 12 and Supplementary Fig. 13).

The cost implications of the additional energy use for adaptation on households and economic activities are unequal between world regions. Annual per capita ESC will increase by 105 $/person on average across years and regions in the current policy scenario. The regions that will experience an increase in the per capita ESC above (below) the world average include the USA, MENA, South-East Asia and Indonesia (Canada, China, India, and Europe). A similar absolute increase in the per capita ESC has different implications between middle- and high-income countries. While in the US an increase of over 310 $/person accounts for a share of 0.4% of the regional per capita GDP, in the MENA region, an increase of 250 $/person accounts for more than 0.7% of the regional per capita GDP (see Supplementary Fig. 9).

### Implications on emissions and global carbon prices

Energy needs for adaptation induce variations in the energy markets that ultimately result in a shift in global and regional greenhouse gas (GHG) emissions. In the current policy scenario, cumulative GHG adaptation emissions reach 350 GtCO_2_eq by the end of the century, accounting for about 7% of the total cumulative GHG emissions from 2020 to 2100 (Supplementary Fig. 10).

**Fig. 6:**
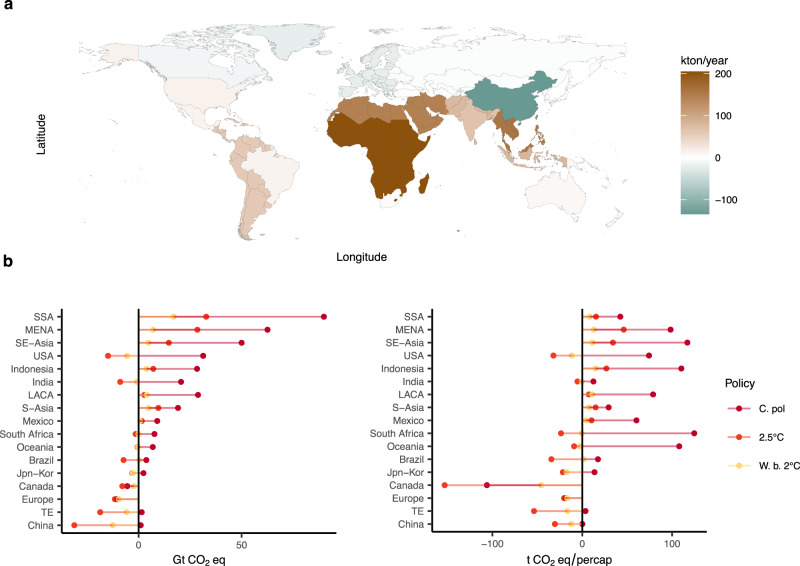
Regional variation in greenhouse gas (GHG) emissions and air-pollutants. **a** Variation in air-pollutants by region. Average total annual increase between 2020 and 2100 in the following air-pollutants: black carbon (BC), nitrogen oxides (NO_*x*_), carbon monoxide (CO), sulphur dioxide (SO_2_), organic compounds (OC), volatile organic compounds (VOC). **b** Additional cumulative GHG emissions for adaptation in 2100, total (left) and per capita (right). Scenarios: Current policies (C.Pol), 2.5 °C and well-below 2 °C (W.b. 2 °C).

The regional distribution of emissions in the current policy scenario reflects the energy mix and the direct shocks in energy demand. In developing and tropical regions, the higher energy needs for adaptation are coupled with a slower energy transition, and therefore additional cumulative emissions are larger than in developed temperate regions. Sub-Saharan Africa ("SSA") accounts for the highest additional cumulative emission increase due to energy for adaptation, but for a comparatively low level of additional cumulative emissions per capita. On the other hand, in regions such as South-East Asia ("SE-Asia") and Indonesia, the additional cumulative emissions are associated primarily with high emissions per capita. The US is the only OECD region where adaptation considerably increases global cumulative GHG emissions (Fig. 6a) in the current policy scenario. Emissions are reduced in countries where the net reduction in energy demand prevails (Europe and Canada).

In the stringent mitigation scenarios, changes in regional emissions compensate each other by virtue of the constraint on the global carbon budget. When a global carbon tax is introduced, emissions are reduced the most in countries with relatively lower marginal abatement costs—e.g., China, Eastern Europe and Russia ("TE"), USA, Brazil, India. The magnitude of the reduction depends on the energy mix and on the extent of the abatement.

The lock-in of additional energy requirements into fossil-based generation, especially in the short-term, has direct consequences not only on GHG emissions but also on air quality (see Fig. 6b). We project a significant increase mainly in nitrogen oxides (NO_*x*_), carbon monoxide (CO), and sulfur dioxide (SO_2_), three of the key air-pollutants related to the combustion of coal and oil^45,46^ (see Supplementary Fig. 11). Average annual emissions of air-pollutants have their peak rise in Sub-Saharan Africa, South-East Asia, and MENA, increasing by about 200, 157, and 145 kton/year, respectively. Although the high level of spatial-temporal aggregation poses challenges to the identification of health impacts, our results suggest that people’s exposure to high levels of pollution increases due to the adaptation-energy feedback, especially in low- to middle-income countries^47,48^. We leave for further research the quantification of health costs related to the additional emissions of air-pollutants and the analysis of how outcomes can be influenced by alternative narratives on technological change, efficiency improvements, and policies directed at pollution control.

As a consequence of the variation in GHG emissions, the adaptation-energy feedback affects the level of the global carbon price needed to achieve the desired carbon budget (Table 4). The carbon price increase is highest in the least ambitious scenarios, as it grows by up to 30%, corresponding to a 5–8 (13–21) $/tCO_2_eq increase in 2050 (2100), while it increases by 5% in the most ambitious mitigation scenarios ("Well-below 2 °C" scenario).

**Table 4:** Carbon tax ($/ton CO_2_ eq.)

Year	2.5 °C		W.B. 2 °C	
	w/o Ada	w/ Ada	w/o Ada	w/ Ada
2030	8	10 (+31%)	74	78 (+5%)
2050	16	21 (+31%)	151	158 (+5%)
2100	44	57 (+30%)	422	443 (+5%)

## Discussion

Integrating climate change impacts and adaptation in energy scenarios contributes to a more accurate understanding of mitigation scenarios and the energy transition^49^. This paper provides an account of how energy use for adaptation can endogenously affect mitigation goals and the design of cost-effective mitigation policies. Since the adaptation-energy feedback increases the energy system costs, our integrated framework captures mitigation’s benefits in terms of reduced adaptation needs, reinforcing previous findings from aggregate macroeconomic assessments^50^. Our simulated net increase in the global energy demand of residential and commercial buildings for adaptation confirms the literature’s finding that the energy demand of buildings is underestimated when IAMs rely solely on income and population drivers and disregard changing climatic conditions^30^ (see Supplementary Fig. 6 and Supplementary Fig. 7). Here, we show that broadening the sectorial scope can provide relevant insights with respect to the assessments that focused on the buildings sector^30^. When the industrial sector is accounted for, the net additional energy needs for adaptation in 2100 under the current policy scenario are more than three times larger than when only buildings are considered (85 and 25 EJ/year, respectively).

The supply-side impacts found in this study can be compared to a narrow set of model-based assessments conducted for the United States: we project a +5% increase in power generation, fuel, and grid costs in the United States under the current policy scenario by 2050, in line with the estimates by ref. 51, and a 20% increase in total installed capacity in 2050 under the current policy scenario, in line with the 16% increase found by ref. 52 under the RCP 8.5. We expand from the literature by quantifying the global additional investments required to transform the energy system to accommodate the energy use for adaptation. We find that the additional energy use for adaptation is largest in South-East Asia and Africa, highlighting the risk that existing vulnerabilities may be further exacerbated if power systems are poorly prepared to face the additional power demand for key services such as air-cooling.

If households and industries use more energy to cope with the ongoing and expected changes in climate conditions, the mitigation challenge can look inherently different. In a scenario where the ambition of mitigation policy does not rise rapidly, climate adaptation contributes to further exacerbate the risk of lock-in into polluting fossil-fuel-based generation in the next few decades. The additional final energy demand and the resulting energy costs are cut by 50% when aiming at the 2.5 °C targets and by up to 75% when reaching the target of Well Below 2 °C. Nevertheless, even in the well-Below 2 °C target, an additional 10 EJ (20 EJ) of energy demand would be required annually by 2050 (2100). If power is not fully decarbonized, by 2050 adaptation could need an average annual addition of new fossil-fuel capacity of about 55 GW, which corresponds to around 1% of the currently installed global fossil-based capacity and is comparable to the new coal capacity added yearly between 2017 and 2021 and to the global new investment decisions for gas-fired generation in 2019^41,53^. As a consequence, energy system costs and carbon prices increase because of adaptation.

Ignoring the energy system costs and the environmental implications of rising adaptation needs in IAMs results in an overestimation of the relative costs of ambitious mitigation policies. Developing scenarios that gather more evidence on the positive side-effects of mitigation policies can help accelerate the tightening of the emission reduction targets within the framework of the Paris Agreement. The potential tension between mitigation and adaptation would be much more significant if the integrated approach proposed in this paper were expanded to include other mechanisms through which responses to climate change affect energy demand^4^, such as water supply and treatment, transportation, and cooling chains, and if the welfare and well-being implications of both adaptation and residual damages were considered. Mitigation can reduce the health costs associated with carbon-intensive adaptation since additional fossil-fuel-fired generation contributes to air pollution. Although empirical estimates on adaptation benefits are growing^54,55^, they remain difficult to be included in IAMs.

While this paper has the ambition to shed light on one type of interaction between mitigation and adaptation at the global scale, several caveats remain. On the one hand, the way we characterize the responses of energy demand to meteorological conditions could actually lead to an overestimation of climate change impacts. We implicitly assume that energy demand does not significantly respond to daily temperatures between 12.5 °C and 27.5 °C. Accounting for the nonlinear response of energy demand across the full distribution of daily temperatures would make it possible to factor in the attenuating impact of fewer moderate temperature days, reducing the energy demand shocks. In the same direction, behavioral changes related to the utilization of heating and cooling appliances^14^ and new business practices, including greater consumer autonomy, digitalization, and new consumer-driven business models^56^, could contribute to lowering the energy requirements of adaptation. The behavioral adjustments simulated in this study can be more directly related to autonomous forms of adaptation undertaken independently by final users. In the long term, we can expect planned adaptation strategies, including, for example, passive cooling, reflective roofs, and urban greening^57^, to become more common. More energy-efficient buildings in the global North, and better performing new residential buildings in the global South, could significantly reduce the energy requirements needed to adapt to extreme temperatures^18,56^.

Conversely, adopting regional-specific thresholds in the computation of extreme climate indices, or accounting for the exacerbation in thermal-discomfort humidity, are aspects that could result in amplifying the additional energy demand we project, especially in tropical regions^58^. Moreover, power system costs projected in this study can underestimate future impacts if peak electricity demand is more sensitive to extreme temperatures than total electricity demand^59^. New empirical evidence on the role that temperature extremes pose to the peak load, rather than on total electricity demand, would contribute to improving the estimation of the potential power system costs induced by climate change adaptation. Future work could explore the costs of an increase in the peak load due to more cooling needs at fine temporal scales by soft-linking global Integrated Assessment Models to bottom-up power capacity expansion and optimal dispatch models. Power generation and transmissions are also vulnerable to climate change (see refs. 60, 61 for a review), and therefore fully characterizing the interaction between mitigation and adaptation requires integrating demand-side and supply-side impacts.

## Methods

### The IAM approach

WITCH is a dynamic global model that fully integrates a simplified representation of the economy, the energy system, and the climate system. The economy is modeled through an intertemporal optimal growth model. A representative agent chooses consumption to maximize regional welfare, and consumption decisions are related to investment choices. The energy sector is hard-linked with the rest of the economy. Energy investments and resources are chosen optimally together with the other macroeconomic variables. Energy demand and, in particular, fuel and technology choices are optimized intertemporally, under a set of constraints, including carbon and other energy prices. A climate model (MAGICC) computes the future changes in the global average temperature on the basis of the GHG emissions generated by economic activities and the energy system. A fully-integrated module translates regional GHG emissions into global temperature through atmospheric concentrations. Another module links the global average temperature increase to changes in regional average temperature based on the linear statistical downscaling model of country-level mean temperature estimated by using future warming scenarios (Representative Concentration Pathways, RCPs, see Section 1 in the Supplementary Methods). WITCH integrates an air pollution module, FASST(R). It is a source-receptor model based on the TM5-FASST model developed by JRC-Ispra, that computes the annual concentrations of several pollutants, namely Sulfur Dioxide (SO2), Nitrogen Oxides (NOx), fine Particulate Matter (PM 2.5), and ground-level Ozone (O3). The fine PM 2.5 concentrations include Particulate Organic Matter (POM), secondary inorganic PM, dust, and sea salt. The FASST(R) model produces concentrations on a world spatial grid of resolution of one degree by one degree and has previously been used to assess premature death from air pollution exposure^62,63^.

### Modeling advancements

Regarding the adaptation–energy feedback loop, a set of equations links the occurrence of extreme temperatures to energy demand. We match the energy demand shocks in WITCH to the available empirical evidence from ref. 31 and therefore use Extreme Temperature Indicators (ETIs) defined as the yearly count of days in which average temperatures fall above the threshold of 27.5 °C and below the threshold of 12.5 °C, respectively. We exclude the moderate temperature intervals and aggregate adjacent extreme bins, and focus on the two temperature intervals of exposure to extreme heat and cold (T < 12.5 °C and T > 27.5 °C).

The heterogeneous relationship between the vector of ETIs (***η***_***i,t***_) and temperature across climate conditions is captured by grouping countries in clusters (Supplementary Methods). We use a polynomial function (*f*) of yearly mean temperatures (*T*_*i*,*t*_). We estimate a panel, fixed-effect model with ordinary least square (OLS) on yearly, country-level observations for 180 countries from 1970 to 2010 (Supplementary Methods). The regional future realizations of the ETIs are then determined endogenously within the model and defined for climatic clusters as follows, *c*, as:

1 $${{{{{{{{\boldsymbol{\eta }}}}}}}}}_{i,t}=f({T}_{c\in i,t},{T}_{c\in i,t}^{2})$$

where

*i* regions (17 regions)

*c* clusters (4 clusters)

*t* 5-year time step in the model from 2005 to 2100

Sector-specific, semi-elasticities are used to link energy demand and ***η***_***i,t***_. They are calibrated after the estimates published by^31^, which model the long-term relationship between energy demand, weather, income, and prices as a dynamic adjustment process. Semi-elasticities indicate the percentage by which demand shifts relative to its conditional mean level in consequence of an additional day occurring in a given interval (j) with respect to the reference temperature interval. The semi-elasticities are specific to two macro-regional groups: temperate and tropical countries. In both macro-groups, the number of days falling within the extreme temperature intervals lies in the tails of the daily temperature distribution (Supplementary Fig. 2 presents a stylized representation of the energy demand shock based on the two extreme temperature bins in tropical and temperate countries). The semi-elasticities provided by ref. 31 capture how energy responds to long-term weather shocks, allowing us to project future energy demand shocks that account for extensive margin adjustments (e.g., purchase of air conditioners, improvements in energy efficiency). Other appealing features of the analysis developed in ref. 31 are that it captures the potential nonlinearity in the demand responses to weather and climate, provides asymmetric responses in temperate and tropical countries, and separates the influence of humidity and temperature on demand. The lack of empirical evidence providing alternative demand response functions for multiple fuels, sectors of the economy, and climate areas limits the scope for assessing the robustness of the results based on ref. 31. The transmission of the climate shock in the commercial and industrial sectors in tropical economies reflects the extensive use of distributed petroleum-fired generators to satisfy final electricity demand.

Sectorial semi-elasticities (*β*_*i*,*f*,*s*,*j*_) are aggregated with the share of the final energy demand of each sector over the total final energy demand as weights (*λ*_*i*,*f*,*s*,*t*_), for each fuel and for each time step of the model. The share is computed from the baseline model projections in each 5-year time step. The aggregation yields a set of semi-elasticities ${\overline{\beta }}_{i,f,t,j}$ specific to each region (*i*), energy vector (*f*), and year (*t*).

2 $${\overline{\beta }}_{i,f,t,j}=\mathop{\sum}\limits_{s}{\lambda }_{i,f,s,t}{\beta }_{i,f,s,j}$$

where

*i* regions (17 regions)

*t* time step in the model, 2005–2100

*f* energy vector (electricity EL, nonelectric energy GAS, and OIL)

*s* sectors (residential, commercial, and industrial)

*j* average daily temperature interval

Climate-induced shocks on energy demand, (Φ_*f*,*i*,*t*_), combine historical and future realizations of the ETIs with average sectorial semi-elasticities aggregated over the two temperature intervals (*j*):

3 $${{{\Phi }}}_{i,f,t}=\frac{\exp ({\sum }_{j}\;{\overline{\beta }}_{i,f,t,j}{{{{{{{{\boldsymbol{\eta }}}}}}}}}_{{{{{{{{\boldsymbol{i,t}}}}}}}}})}{\exp ({\sum }_{j}\;{\overline{\beta }}_{i,f,j}{{{{{{{{\boldsymbol{\eta }}}}}}}}}_{{{{{{{{\boldsymbol{i,t}}}}}}}}})}-1$$

where

*i* regions (17 regions)

*t* time step in the model, 2005–2100

*f* energy vector (electricity EL, nonelectric energy GAS, and OIL)

*j* average daily temperature interval

We follow ref. 64 and assume that the climate-induced energy demand shocks affect the productivity of the energy inputs entering into the aggregate production function. If climate-induced shocks increase energy demand, it is as if the economic systems needed more energy to produce output. Climate-related positive shocks (i.e., increase in energy demand) are therefore modeled as technological retrogression, requiring more inputs to generate a given output. In the WITCH model, energy (EN) is a combination of electricity (EL) and nonelectric energy (NEL), which includes coal, gas, and oil. Electricity and nonelectric energy can be substituted with an elasticity of substitution, *ρ*_*EN*_:

4 $${{{{{{{{\rm{EN}}}}}}}}}_{i,t}={[{\tilde{\alpha }}_{{{{{{{{\rm{EL}}}}}}}},i}{{{{{{{{\rm{EL}}}}}}}}}_{i,t}^{{\rho }_{{{{{{{{\rm{EN}}}}}}}}}}+{\tilde{\alpha }}_{{{{{{{{\rm{NEL}}}}}}}},i}{{{{{{{\rm{NEL}}}}}}}}{i,t}^{{\rho }_{{{{{{{{\rm{EN}}}}}}}}}}]}^{\frac{1}{{\rho }_{{{{{{{{\rm{EN}}}}}}}}}}}$$

In this formulation, the productivities of electricity and nonelectricity are endogenous functions of climate shocks:

5 $${\tilde{\alpha }}_{{{{{{\mathrm{EL}}}}}},i,t}={\alpha }_{{{{{{\mathrm{EL}}}}}},i}\frac{{{{\Phi }}}_{{{{{{\mathrm{EL}}}}}},i,t}{Q}_{{{{{{\mathrm{EL}}}}}},i,t}}{{\sum }_{f}{Q}_{f,i,t}}$$

6 $${\tilde{\alpha }}_{{{{{{\mathrm{NEL}}}}}},i,t}={\alpha }_{{{{{{\mathrm{NEL}}}}}},i}\left[\frac{{{{\Phi }}}_{{{{{{\mathrm{GAS}}}}}},i,t}\;{Q}_{{{{{{\mathrm{GAS}}}}}},i,t}}{{\sum }_{f}\;{Q}_{f,i,t}}+\frac{{{{\Phi }}}_{{{{{{\mathrm{OIL}}}}}},i,t}\;{Q}_{{{{{{\mathrm{OIL}}}}}},i,t}}{{\sum }_{f}\;{Q}_{f,i,t}}\right]$$

### Quantification of additional new capacity

In the WITCH model, investments in new power generation plants to fulfill electricity demand depend on: (i) the cost of electricity generation of the different technologies, which combines capital costs, Operation and Maintenance (O&M) expenditure, and the costs for fuels in an endogenous way; (ii) the lifetime power plants; (iii) a constraint on the flexibility of the power generation fleet to accommodate the integration of renewables; (iv) an installed capacity constraint on the power generation fleet to guarantee that sufficient capacity is built to meet the instantaneous peak electricity demand (for further details see ref. 37).

The cumulative additional new capacity added in response to the variation in electricity demand required for adaptation that we report (Γ_*h*,*i*,*t*_) for each technology *h* in region *i* at time *t* is computed as follows:

7 $${{{\Gamma }}}_{h,i,t}=\mathop{\sum }\limits_{t=2005}^{t}\left({K}_{h,i,t}^{{{{{{\mathrm{Ada}}}}}}}-{K}_{h,i,t}^{{{{{{\mathrm{NoAda}}}}}}}\right)$$

8 $${K}_{h,i,t+1}={K}_{h,i,t}{((1-{\delta }_{h,i,t+1}))}^{{{{\Delta }}}_{t}}+{{{\Delta }}}_{t}\frac{{I}_{h,i,t}}{S{C}_{h,i,t}}$$

Where *δ*_*h*,*i*,*t*+1_ is a depreciation rate based on a finite lifetime of the power plant, *I*_*h*,*i*,*t*_ are the annual investments, and *SC*_*h*,*i*,*t*_ the investment cost.

### Quantification of energy costs

Power generation costs (C_GEN), include the investments in generation capacity (I), R&D investments in power generation technologies (I_RD), O&M costs (OM), and fuel expenditures for power generation (E_FUEL):

9 $${{{{{{{{\rm{CGEN}}}}}}}}}_{{{{{{{{\rm{i,t}}}}}}}}}=\mathop{\sum}\limits_{h}({{{{{{{{\rm{I}}}}}}}}}_{{{{{{{{\rm{h,i,t}}}}}}}}}+{{{{{{{{\rm{I}}}}}}}}\_{{{{{{{\rm{RD}}}}}}}}}_{{{{{{{{\rm{h,i,t}}}}}}}}}+{{{{{{{{\rm{OM}}}}}}}}}_{{{{{{{{\rm{h,i,t}}}}}}}}}+{{{{{{{{\rm{E}}}}}}}}\_{{{{{{{\rm{FUEL}}}}}}}}}_{{{{{{{{\rm{h,i,t}}}}}}}}})$$

where

*i* regions (17 regions)

*t* time step in the model, 2005–2100

*j* power generation technology

Fuel costs (C_FUEL) include the investments and O&M costs in fossil-fuel extraction (OM_ex) and the expenses associated with liquids and gas consumption (EXP_ff), excluding the expenses related to fuel consumption in the power sector:

10 $${{{{{{{{\rm{C}}}}}}}}\_{{{{{{{\rm{FUEL}}}}}}}}}_{{{{{{{{\rm{i,t}}}}}}}}}=\mathop{\sum}\limits_{f}({{{{{{{{\rm{OM}}}}}}}}\_{{{{{{{\rm{ex}}}}}}}}}_{{{{{{{{\rm{i,t,f}}}}}}}}}+{{{{{{{{\rm{EXP}}}}}}}}\_{{{{{{{\rm{ff}}}}}}}}}_{{{{{{{{\rm{i,t,f}}}}}}}}})$$

where

*i* regions (17 regions)

*t* time step in the model, 2005–2100

*f* fuel

Investments in the electrical grid (I_GRID) are computed based on grid capital. The grid capital stock is adjusted by taking into account a linear relationship between grid capacity and the capacity of traditional power generation technologies and the investments for integrating the generation of variable renewables. A detailed description is available in ref. 37.

### Scenarios

In the current policy scenario, GHG emission targets extrapolate beyond 2020 the implied ambition levels of current climate policies until 2020. Overall, the current policy scenario with no energy-adaptation feedback leads to cumulative carbon emissions of about 5000 GtCO_2_eq, from 2018 until 2100 Table 5. More stringent mitigation scenarios keep the increase in global mean temperature in 2100 at 2.5 °C and well below 2 °C, resulting in cumulative GHG emissions from 2018 until 2100 of 3600, and 1500 GtCO_2_eq, respectively. Non-CO_2_ greenhouse gases in these scenarios are priced equivalently to the implied CO_2_ prices, by using 100-year global warming potentials for conversion. We use explicit GHG pricing, and climate stabilization targets are achieved in a global cost-optimal way, with no international compensation scheme or carbon emission trading.

**Table 5:** Climate scenarios assessed in this study

Scenario	Fixed carbon budget	Carbon emissions (2018–2100)	Global mean temperature increase (2100)
Current policy	No	5000 GtCO_2_eq	3.23 °C
2.5 °C	Yes	3600 GtCO_2_eq	2.56 °C
Well-below 2 °C	Yes	1500 GtCO_2_eq	1.65 °C

Population^65^ and country-level GDP projections implemented by using Purchasing Power Parities (PPP)^66^ are based on the basic and extended SSPs^40^. The main results use the Shared Socio-Economic Pathway Middle-of-the-Road (SSP2), which is a continuation of the historical trends, while the SI presents some results across SSPs. For more information on the implementation of key aspects, such as energy productivity, land-use and power technologies, and fossil-fuel resources, see ref. 37.

## Supplementary information

Supplementary information

## Data Availability

The output data used to perform the analysis and the R-scripts used to produce the figures are available in the repository: 10.5281/zenodo.6838739^67^. The full set of output data are available from the corresponding author on reasonable request. Additional raw input data used in this analysis are available at the following public locations: NASA/NOAA GLDAS: [https://disc.gsfc.nasa.gov/datasets/GLDAS_CLSM025_D_2.0/summary];

## Code availability

The code building the additional WITCH modules and the input data generated in this study are available in the repository: 10.5281/zenodo.6838739^67^. The results obtained in this study can be replicated by running the additional WITCH modules and input data in combination with the open source WITCH available in the repository: https://github.com/witch-team/witchmodel.
