# Supplementary material for: Increased energy use for adaptation significantly impacts mitigation pathways
Source: Nat Commun. 2022 Aug 24;13:4964. doi: 10.1038/s41467-022-32471-1 (PMC9402953; doi:10.1038/s41467-022-32471-1)
Supplement: Supplementary file 1 — Supplementary information [file 41467_2022_32471_MOESM1_ESM.pdf]

Supplementary Information to: Increased energy use for adaptation significantly impacts mitigation pathways

Francesco Pietro Colelli*[1,2], Johannes Emmerling[3], Giacomo Marangoni[3,4], Malcolm Mistry[1,5], Enrica De Cian[1,2,3]

[1] Department of Economics, Ca' Foscari University of Venice, 30121 Venice, Italy

[2] Fondazione Centro Euro-Mediterraneo sui Cambiamenti Climatici (CMCC), 30175 Venice, Italy

[3] RFF-CMCC European Institute on Economics and the Environment (EIEE), Fondazione Centro Euro-Mediterraneo sui Cambiamenti Climatici, 20144 Milano, Italy

[4] Department of Economics, Management and Industrial Engineering, Politecnico di Milano, 20156, Milan, Italy

[5] Department of Public Health, Environments and Society, London School of Hygiene Tropical Medicine, WC1H 9SH, London, United Kingdom

Email address of corresponding author: francesco.colelli@unive.it

Supplementary Figures

Regional exposure to extreme temperatures

Supplementary Figure 1 shows the future changes in the frequency of warm and cold days across regions with respect to the model's projection for the begin of the time period (2005). Within the 17 regions included in the WITCH model, Indonesia, South-East Asia and Sub-Saharan Africa experience the largest increase in annual warm days (reaching up to 100 additional warm days), while Europe, the Middle East and the United States experience the largest decrease in the number of cold days.

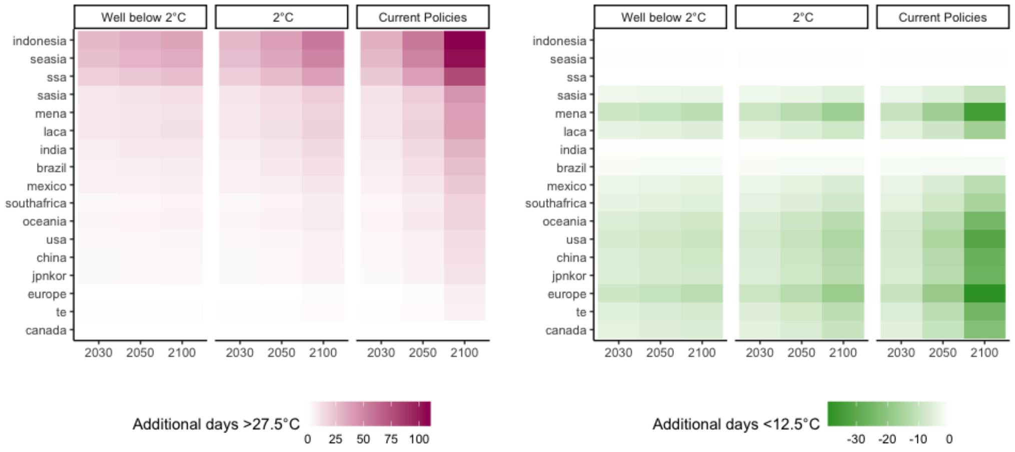

Supplementary Figure 1: Future changes in the frequency of warm and cold days. Difference in the occurrence of warm (left) and cold (right) days in the four future scenarios for the 17 regions of the WITCH model around 2030 (2020-2040 average), 2050 (2040-2060 average) and 2100 (2090-2110 average) compared to the model's projected value in 2005.

Final energy demand for adaptation

Supplementary Figure 2 shows the future energy requirements of adaptation in buildings (residential and commercial sectors), excluding industry which is shown in the main paper. An additional 6 EJ (25 EJ) of final energy is projected in 2050 (2100). Higher electricity demand is only partially compensated by lower final demand for liquids and gas (Supplementary Figure 2, panel a). In relative terms, such requirements amount to a 5% (12%) scale up of final electricity demand and -1% (-3%) decrease of liquids and gases demand by 2050 (2100). Setting ambitious mitigation

targets lowers the pressure on buildings' power demand considerably, but not completely as adaptation needs would still require more electricity: from 17 EJ in the "Well below 2°C" to a 33 EJ in the "2.5°C" scenario. The decrease in liquids and gas demand on the other hand becomes negligible in the most ambitious mitigation scenario while is around -10 EJ in 2100 in the "2.5°C" scenario.

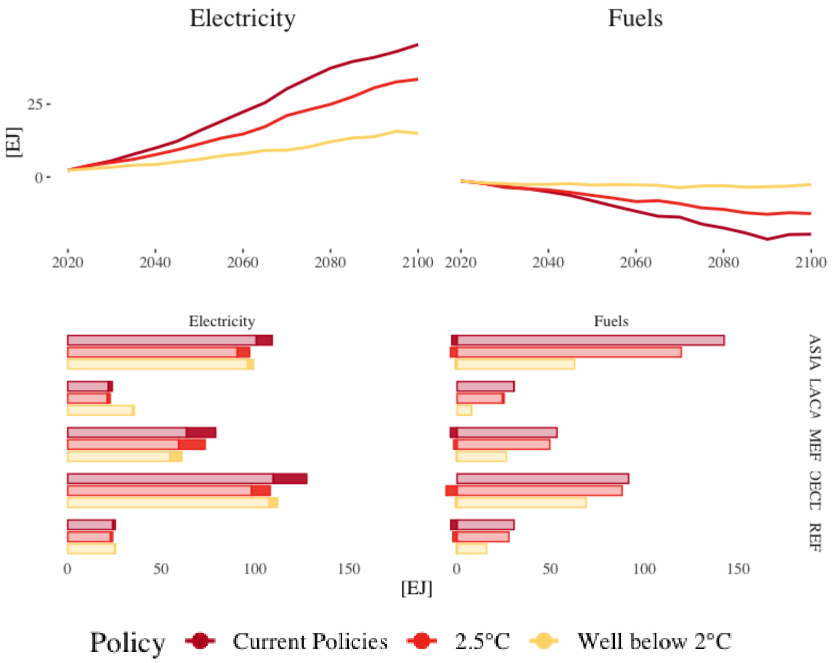

Supplementary Figure 2: Projected energy for adaptation demand of electricity (left) and liquid and gas fuels (right) in the buildings sector under the SSP 2. Panel a shows the yearly increase in global demand from 2020 to 2100 across the different scenarios. Panel b shows the regional demand in 2100 without adaptation (light shaded bars) and the additional demand required for adaptation (dark shaded bars) under the SSP 2. Labels in panel b show the regional percentage increase of final energy demand induced by the adaptation-energy feedback. The additional demand of liquids and gas includes both traditional fossil fuels and bio-fuels, as the latter are substitutes of the former.

New power capacity requirements

The additional pressure posed on power generation by the increase in electricity demand results in a scale-up of both fossil-based and renewables capacity, as well as of storage capacity. The additional new generation capacity required in the next few decades for adaptation comprises both fossil-based and renewable generation. Renewables and storage constitute most of the additional new capacity in the second half of the century across all scenarios (Supplementary Figure 4, panel a). The share

of the additional coal, oil and gas capacity on the total additional capacity required to fulfill adaptation needs (Panel b) ranges from 85% (current policies) to 80%-65% (ambitious mitigation scenarios) in 2030, from 40% to 20%-10% in 2050 and up to 10% in 2100 in the current policies.

We measure the annual long-term improvement rate of energy intensity (final energy per unit of GDP in PPP in MJ/$) and carbon intensity ($CO_2$ emissions over final energy in gCO2/MJ) as an indication of the energy sectors' transition across climate scenarios (Supplementary Figure 3). The annual long-term improvement rate is computed as the average annual variation from 2020 to 2100. Negative values express a reduction in the energy intensity and carbon intensity. The introduction of stringent mitigation policies leads to concurrent improvements of both the energy intensity of the economy and the carbon intensity of energy (similarly to the multi-model projections in [1]). Both globally and across regions, the annual long-term improvement rates of energy intensity are reduced (become smaller in absolute values) when taking into account the energy requirements for adaptation, while the long-term improvement rates of carbon intensity are mostly unaffected. Supplementary Figure 4 shows the cumulative generation capacity required to meet the electricity demand with (w Ada) and without (w/o Ada) the adaptation feedback, including fossil fuels, renewable energy and storage.

Variation in the energy system costs

Supplementary Figure 5 shows the decomposition of the additional energy supply costs by a unit of total final energy demand. The additional electricity supply costs over total electricity consumption increase sharply from 2-5 USD/MWh in 2030 to up to 10-15 USD/MWh in 2050-2100 under the current policies scenario, while remain stably below 5 USD/MWh over the whole period in the well below 2°C scenario. The unitary additional energy supply costs for fuels remain below 3-4 USD/MWh even in the current policies scenario. Supplementary Figure 6 shows the variation in the Levelized Costs of Electricity (LCOE) due to the adaptation-energy feedback. The LCOE increase ranges between 2 to 3 USD/MWh under the current policies scenario, a 2-7% increase from the baseline LCOE with no adaptation feedback depending on the year and scenario. The highest relative increase is projected for

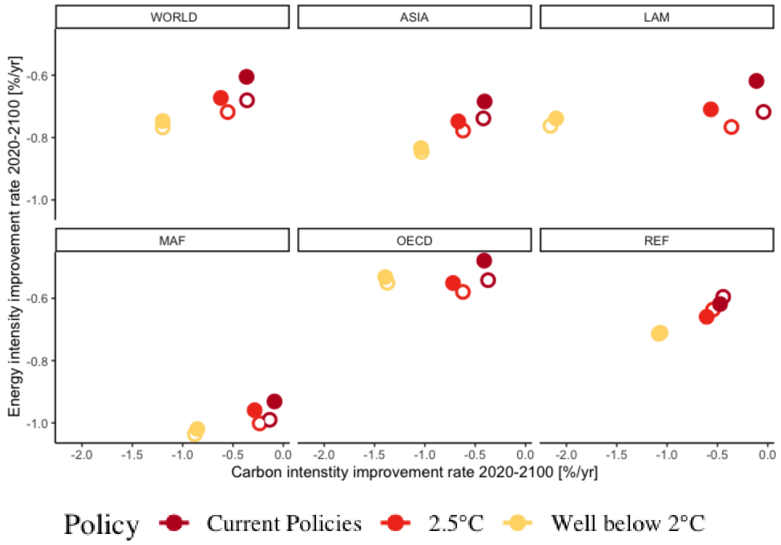

Supplementary Figure 3: Annual long-term improvement rates of energy intensity (final energy/GDP in PPP) and carbon intensity (CO_2/final energy) by scenario and region (Panel a). The development in the scenario with no energy-adaptation feedback (empty markers) is compared to the development in the scenarios comprising the feedback (full markers).

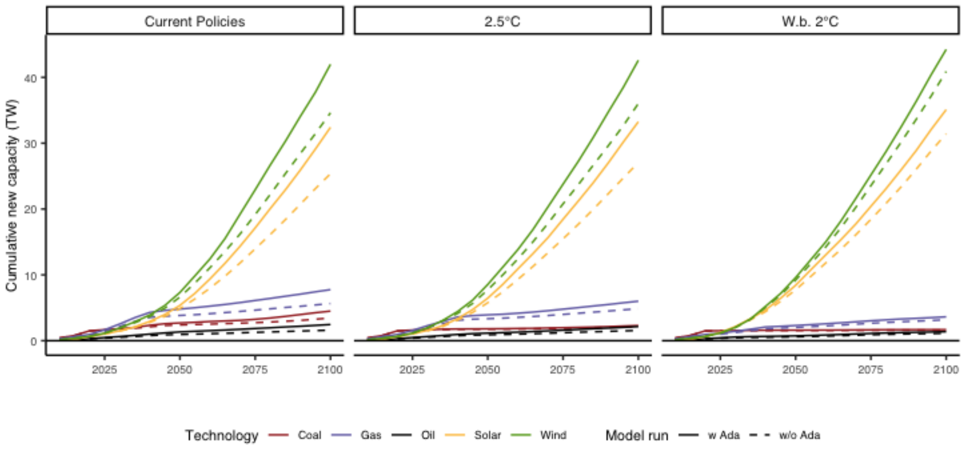

Supplementary Figure 4: Cumulative generation capacity by technology with and without the adaptation feedback. The technologies whose annual additional new capacity was not affected by the adaptation feedback have been excluded.

the current policies scenario around 2100.

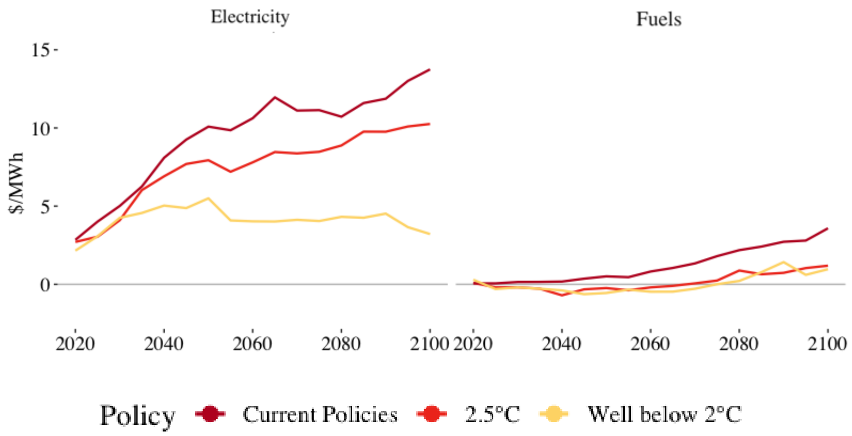

Supplementary Figure 5: Annual additional energy supply costs by unit of total final energy demand for electricity (left) and fuels (right). Additional refers to the additional demand and costs compared to the corresponding scenarios without the adaptation-energy feedback.

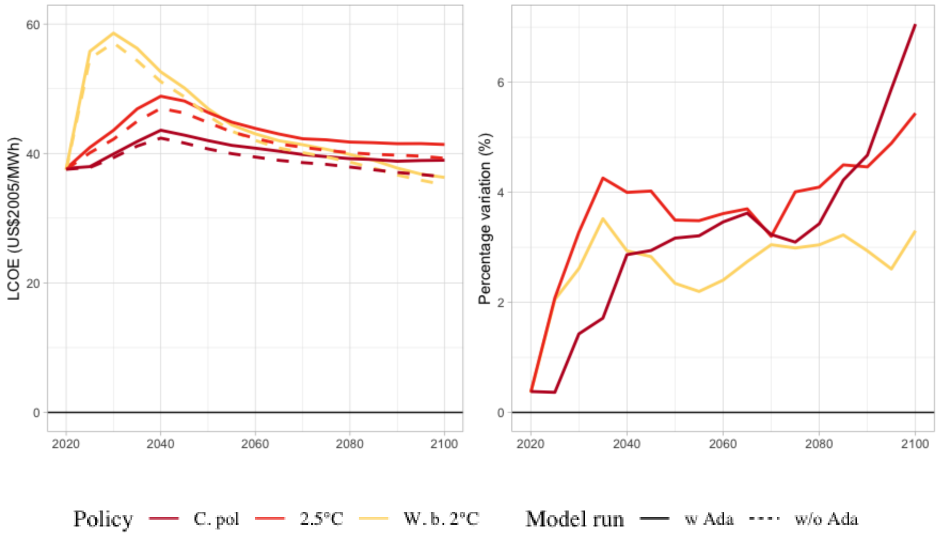

Supplementary Figure 6: Levelized Cost of Electriciy (LCOE) across scenarios. Panel a: yearly LCOE level without and with adpatation feedback. Panel b: yearly percentage change in LCOE due to the adaptation feedback.

Implications on emissions and global carbon prices

Supplementary Figure 8 shows the cumulative emissions of GHG in the three scenarios. Only in the current policies scenario the additional energy for adaptation results in an increase in global cumulative emissions, that reach 350 GtCO_2eq, about

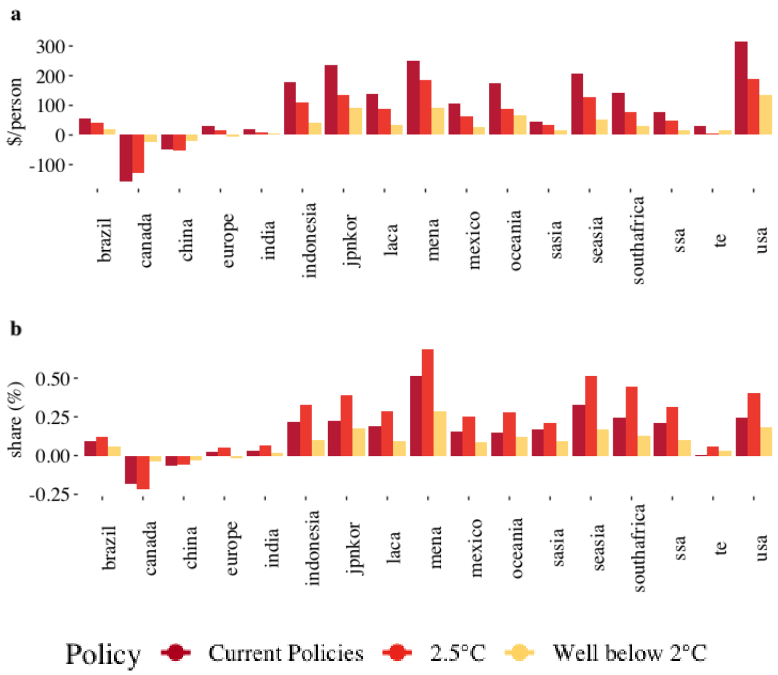

Supplementary Figure 7: Additional energy system costs per capita by region. Panel a: Absolute value of the additional per capita costs. Panel b: Share of the additional per capita costs over the region per capita GDP. Annual average between 2020 and 2100.

7% of the total cumulative GHG emissions from 2020 to 2100. In the other scenarios the global cumulative GHG emissions do not change when the adaptation-energy feedback is accounted for, due to the fixed carbon budget.

Supplementary Figure 9 shows the annual average variation in the emissions of six key pollutants in the three scenarios due to the additional energy production and consumption for adaptation.

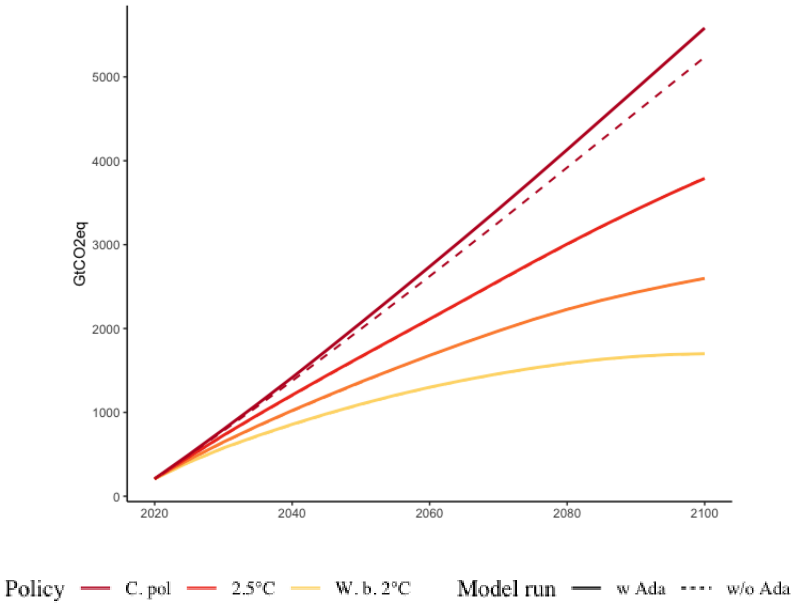

Supplementary Figure 8: Variation in the cumulative GHG emissions. Panel a: Cumulative GHG emissions from 2020 to 2100 with (solid lines) and without (dotted lines) the energy-adaptation feedback.

Results by SSPs

Supplementary Figure 10 presents the incremental energy demand and costs (panel a) and the increase in the carbon tax (panel b) due to climate change in 2100 across SSPs and climate policy outcomes. The impacts on the energy system across socio-economic pathways are scaled uniformly from the lower end of the range in SSP 3 to the higher end in SSP 5. The level of the additional demand and costs in the SSP 5 are roughly two times the levels in the SSP 3, while the moddle-of-the-road SSP 2 lies in between (being closer to the SSP 3 as for the energy costs and the absolute carbon tax increase). The increase in the global average temperature in 2100 of an additional +0.5C, from +2C to +2.5C, strongly affects mitigation policies. While the energy demand, the supply-side costs and the level of the carbon tax increase only marginally, the relative change in the carbon tax goes from a 5%-10% variation to a 20%-30% variation.

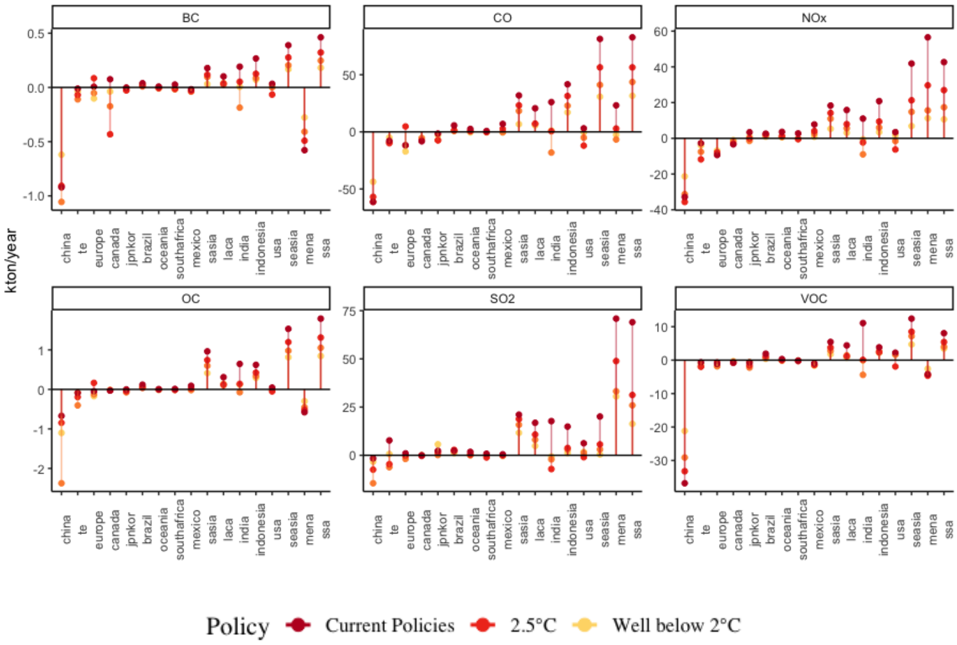

Supplementary Figure 9: Annual average variation in the emissions of black carbon (BC), nitrogen oxides (NOx), $carbonmonoxide (CO)$, $sulphurdioxide (SO_2)$, organic compounds (OC), volatile organic compounds (VOC), across scenarios, due to the additional energy production and consumption for adaptation.

Supplementary Figure 11 shows the variation in the cumulative ESC for power system costs associated to the more ambitious mitigation policy scenarios with respect to the current policies scenario, across SSPs and in the case without and with the adaptation-energy feedback. The Net Present Value (NPV) is computed based on a i.r. of 3%.

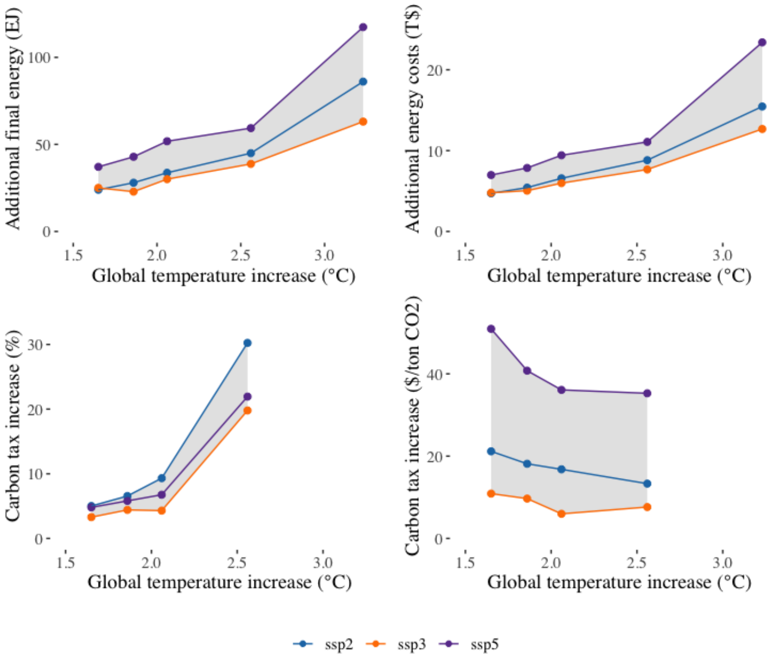

Supplementary Figure 10: Incremental final energy demand, energy system costs, total and relative increase in the carbon tax, due to energy for adaptation in 2100 across SSPs and climate policy outcomes.

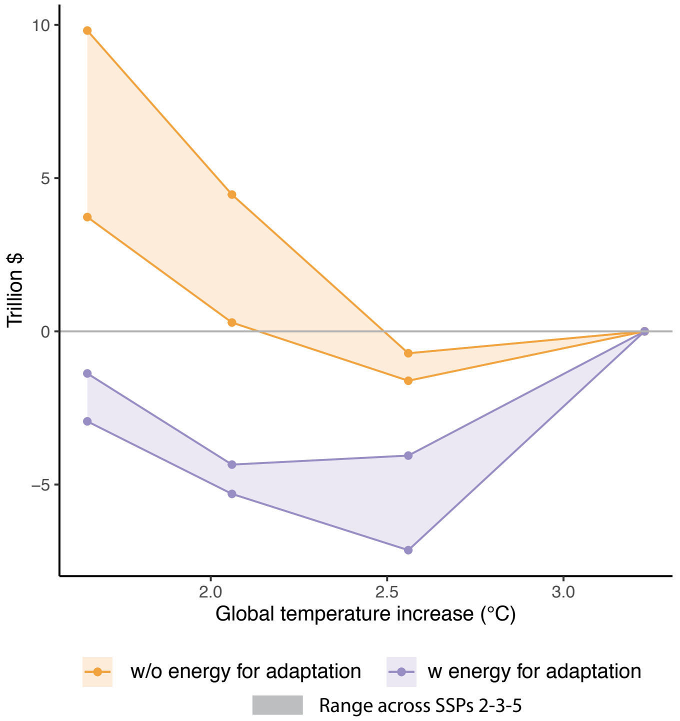

Supplementary Figure 11: Difference in the NPV of ESC for power by scenario (Trillion USD) with respect to the NPV in the current policy scenario. The shaded area represents the range across SSPs.

Supplementary Figure 12 compares our results with the few IAM-based projections providing the variation in global buildings' final energy demand induced by climate change adaptation in 2050 and 2100 [2, 3]. Our projections, ranging from 3 to 5 EJ (12 to 26 EJ) in 2050 (2100) depending on the extent of global warming, are in line with the literature's projected increase under the same socio-economic scenario (SSP2).

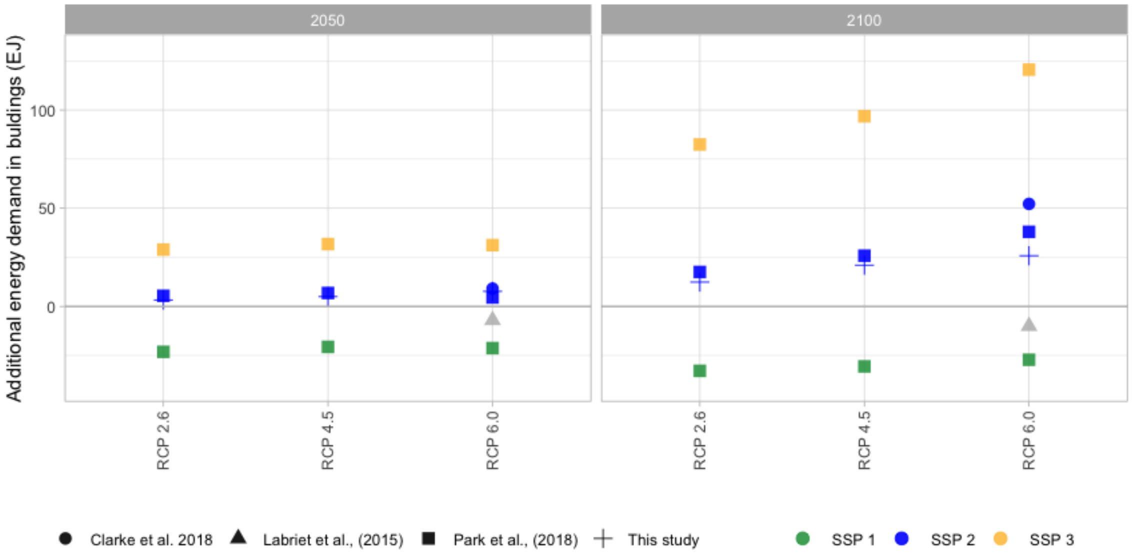

Supplementary Figure 12: Net increase in global buildings' final energy demand across IAMs. The annual increase in 2050 and 2100 is presented across climate change scenarios (RCPs), socio-economic scenarios (SSPs) and models (markers' shape).

Supplementary Figure 13 compares the future energy system costs presented in this study with six leading IAMs' projections reported in [4]. Differently from Figure 4, fuel consumption costs are excluded from the selected energy system costs reported in 13, because [4] only focuses on energy systems' investments. Only comparable energy system costs have been considered, namely: investments in extraction and conversion of fossil fuels, investments in electricity generation and investments in the electricity's transport, distribution and storage. The multi-model median NPV of energy system costs in 2050 (2100), excluding the energy-adaptation feedback, in the current policies scenario is 47 (81) Trillion USD, while we project an NPV of 50 (85) Trillion USD without adaptation and of 54 (95) Trillion USD with adaptation.

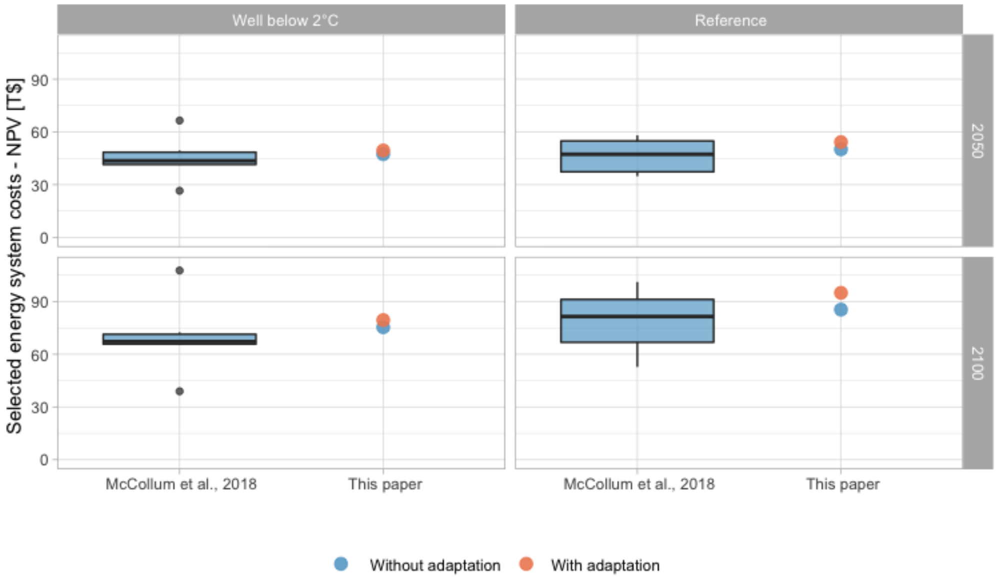

Supplementary Figure 13: Selected global energy system costs. Net Present Value (3% discount rate) of the energy costs incurred from 2015 to 2050 and 2100, including extraction and conversion of fossil fuels, investments in electricity generation and investments in the electricity's transport, distribution and storage. Models included in [4] are: AIM/CGE, IMAGE, MESSAGEix-GLOBIOM, POLES, REMIND-MAgPIE, WITCH-GLOBIOM

Supplementary Methods

The WITCH model

In this section we describe the implementation in the integrated assessment model WITCH [5, 6], which has been used to assess the quantitative magnitude of the effect of climate engineering on the optimal abatement path and a series of key variables of climate mitigation effort. WITCH has been used extensively in the literature of scenarios evaluating international climate policies, for example as a major contributor to scenarios reviewed by the IPCC in its fifth assessment report (`https://tntcat.iiasa.ac.at/AR5DB`). WITCH is a global model with 17 macro-regions. It is a long-term dynamic model based on a Ramsey optimal growth economic engine, and a hard linked energy system which provides a compact but exhaustive representation of the main abatement options, both in the energy and non-energy sectors. The model is solved numerically in GAMS/CONOPT. A description of the model equations can be found on the model website at `http://doc.witchmodel.org` and the general model code is open source and available at `https://github.com/witch-team/witchmodel`.

Country-level temperatures

We used a statistical approach to downscale changes in global average temperature to regional temperature. The statistical downscaling is based on the CMIP5 database [7], which provides historical data and projections of temperature and precipitation at the 0.5° gridded level on an averaged annual basis. We aggregate the data to the country boundaries using population weights, obtaining data for $N = 244$ countries and territories. We use the data from different Representative Concentration Pathways (RCPs) implemented by a number of global climate models and consider the model ensemble mean for all RCPs to link global mean temperature increase (ΔGMT) to the country-level average annual temperature. We run a linear regression to estimate the regional temperature levels in region i at time t denoted as T_{it} (measured in °C) due to the global temperature increase ΔGMT_t based on the

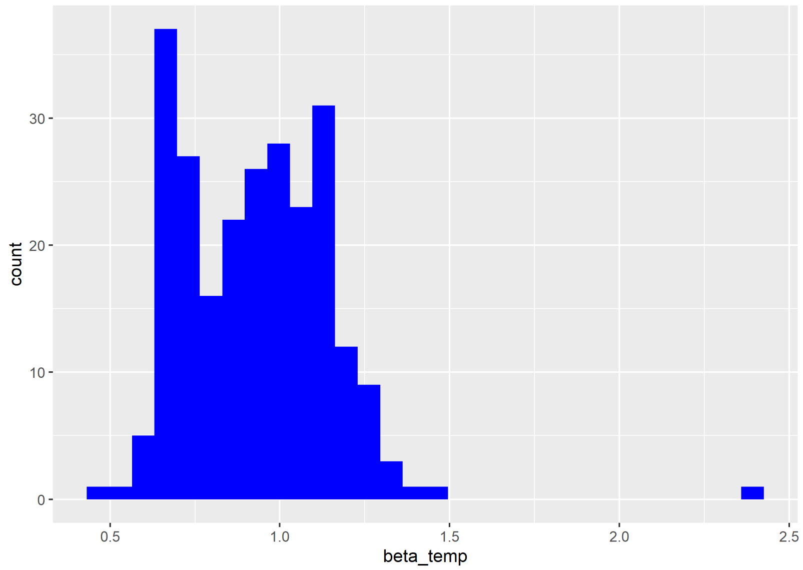

Supplementary Figure 14: Histogram of the estimated $\hat{\gamma}_i$ coefficients describing the relationship between country temperature levels and global average temperature increase.

following equation:

$$T_{it} = \alpha_i^* + \gamma_i \Delta GMT_t + \eta_m \quad (1)$$

The estimated slopes of this relationships ($\hat{\gamma}_i$) are shown in Supplementary Figure 14, which show a homogeneous, single-peaked and thin-tailed distribution, albeit with a significant variance. Based on the regional aggregation of the WITCH model, which considers 17 world regions, we aggregate country-level estimates across regions. Due to the linear relationship, the coefficients used are the population weighted averaged of the estimated coefficients.

Country-level Extreme Temperature Indicators

We empirically estimate a reduced-form relationship between country-level annual average temperatures and the occurrence of extreme cold and warm days in one year. The reduced-form equation is estimated from a panel dataset based on climate reanalysis data covering 180 countries from 1970 to 2010. All weather variables are derived from 3 hourly near surface temperature made available by the NASA Global Land Data Assimilation System (GLDAS) at 0.25 degrees global gridded resolution [8]. Gridded temperature and Extreme Temperature Indicators (ETIs) have been

weighted at the grid-cell level by the share of population over national population. Country level observations have then been computed as the sum of the population weighted variable of each grid-cell. Population data is obtained from the Gridded Population of the World, Version 4 (SEDAC, 2020). We test a model including country fixed effects, controlling for all country specific, time invariant characteristics which may affect the value of the ETIs. In order to capture the heterogeneity in the relation between the ETIs and temperature in counties with markedly different climates, we group the countries by means of a cluster analysis. In our main specification we use the K-means cluster algorithm to conduct the cluster analysis [9], available in R through the "kmeans" function. As a robustness check, we implement also a set of alternative algorithms based on hierarchical agglomerative clustering. Each ETI is evaluated with a separate cluster analysis, based on four variables: yearly temperature, variance of yearly temperature, yearly ETI and variance of yearly ETI. The different methods lead to the identification of a number of clusters varying from three to five for each ETIs. We compare alternative specifications based on standard metrics (adj. R squared, AIC) and select the model based on k-means clustering identifying a maximum number of four different clusters for both ETIs. A dummy variable indicating each cluster is adopted in order to estimate cluster-based polynomial. Different polynomial forms of the relation between temperature and ETIs are tested. The econometric specification tested is the following:

$$y_{i,t} =_1^{(\mathrm{k})} temp_{i,t} d_i^{(\mathrm{k})} + ... +_m^{(\mathrm{k})} temp_{i,t}^{(\mathrm{m})} d_i^{(\mathrm{k})} +_{m+1} c_i + \epsilon_{i,t} \quad (2)$$

where

y climate extreme indices

$temp$ mean temperature

c fixed effects

d cluster dummy variable

ϵ random errors

i countries

t time

m maximum degree of the polynomial

k cluster number (1…n; with n maximum number of clusters)

The combination of three polynomials alternatives (linear, quadratic, cubic) and four maximum number of clusters leads to the estimation of 12 econometric specifications. We estimate the fixed-effect panel model (eq. 8) via OLS. In order to evaluate the robustness of our results, we test for the presence of serial correlation and cross-sectional dependence in our panel. In all cases, the polynomial specifications lead to statistically significant and correctly signed parameters. The results are compared based on standard metrics (adj. R squared, AIC), on the significance of the coefficients robust to both serial correlation and cross-sectional correlation and on the analysis of the normality of the residuals. The results of the specification selected based on such criteria are reported in Table 1.

Supplementary Table 1

	Extreme Temperature Indicator	
	Days <12.5°C	Days >27.5°C
$Temperature$	-2.302*	220.845***
	(1.215)	(74.686)
$Temperature^2$	-0.306***	-2.994**
	(0.068)	(1.386)
$Temperature * Cluster2$	-19.645***	-604.319**
	(4.085)	(241.43)
$Temperature * Cluster3$	-2.044	-225.425***
	(1.519)	(74.688)
$Temperature * Cluster4$	-33.368***	-241.342***
	(6.135)	(75.039)
$Temperature^2 * Cluster2$	0.721***	11.578**
	(0.102)	(1.386)
$Temperature^2 * Cluster3$	-0.347***	3.322**
	(0.094)	(1.386)
$Temperature^2 * Cluster4$	0.817***	4.076***
	(0.167)	(1.386)
$Unitfixedeffects$	Yes	Yes
$AdjustedR^2$	0.495	0.719
$F - test$	900.123***	2,313.281***
N	7,160	7,160

Note: $^{*}p < 0.1$; $^{**}p < 0.05$; $^{***}p < 0.01$

Empirical method for the estimation of the energy demand function

Supplementary Figure 15 presents the stylized response of energy demand for a given sector and fuel combination as a linear spline function based on temperature intervals. In this work we use semi-elasticities provided by [10], that exclude the moderate temperature intervals and aggregate adjacent extreme bins, to focus the analysis on the effects of exposure to extreme hot and cold days ($T < 12.5°C$ and $T > 27.5°C$). The semi-elasticities are specific to temperate and tropical countries. The frequency of the temperature intervals is assessed though the distribution of the population-weighted average daily temperatures from 1970 to 1990 in 174 countries based on GLDAS reanalysis data. In both macro-groups, the number of days falling within the population-weighted temperature interval $> 27.5°C$ lies in the tails of the distribution of historical average daily temperatures. Only 4% and 6% of the historical occurrences of days-country observations fall above the threshold for the temperate and tropical group, respectively. The number of days falling within the temperature interval $< 12.5°C$ is in the tail of the distribution as for tropical countries (4% of the combinations of days-country observations fall below the threshold) while constitute almost the median temperature in temperate countries (45% the of combinations of days-country observations fall below the threshold). The Supplementary Figure 15 shows a stylized set of energy demand responses that are asymmetric and are assumed to exhibit a generalized V-shape, with the slope of each segment capturing the marginal effect on demand of additional exposure to heat or cold. The height of the gray area (Z1 for temperate and Z2 for tropical countries) indicates the magnitude of climate-invariant consumption at the balance point (i.e. the nadir of the V-shape).

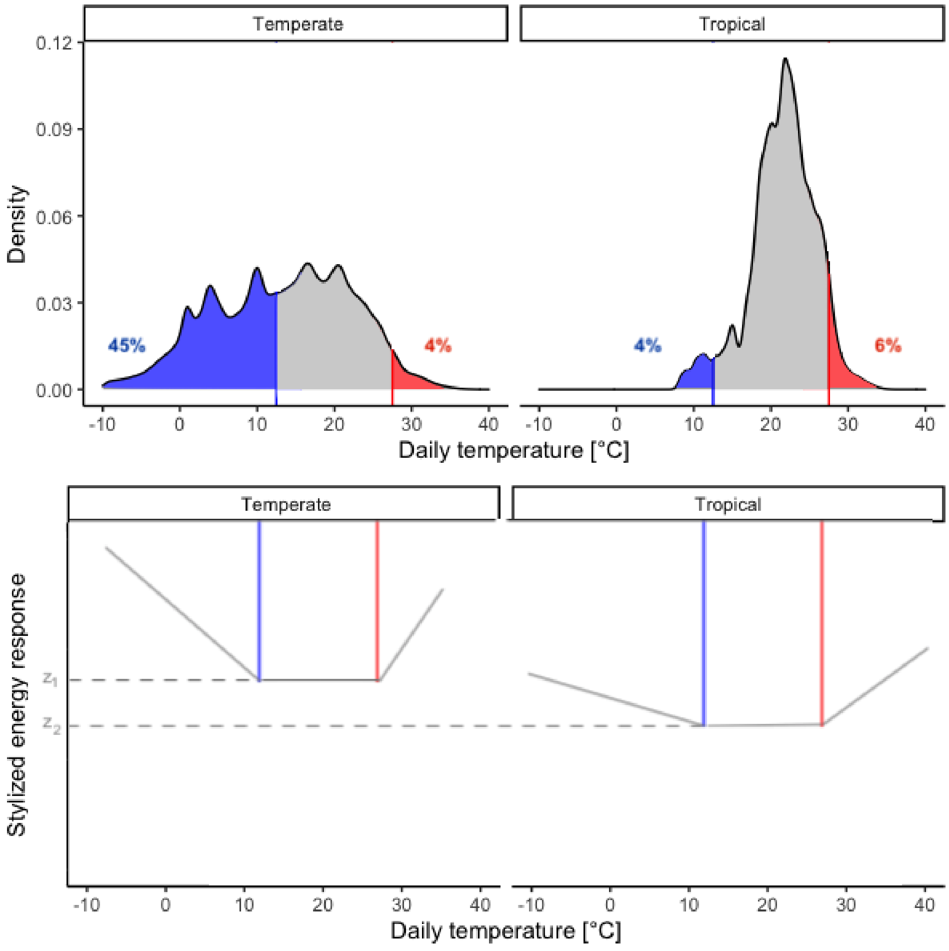

Supplementary Figure 15: Stylized response of energy demand as a linear spline function based on temperature intervals of extreme hot and cold days (T $<$ 12.5°C and T $>$ 27.5°C). The upper panel shows the distribution of population-weighted daily mean temperatures from 1970 to 1990 in 174 countries based on GLDAS reanalysis data.

References

[1] Keywan Riahi et al. "The shared socioeconomic pathways and their energy, land use, and greenhouse gas emissions implications: an overview". In: *Global Environmental Change* 42 (2017), pp. 153–168.

[2] Chan Park et al. "Avoided economic impacts of energy demand changes by 1.5 and 2 C climate stabilization". In: *Environmental Research Letters* 13.4 (2018), p. 045010.

[3] Leon Clarke et al. "Effects of long-term climate change on global building energy expenditures". In: *Energy Economics* 72 (2018), pp. 667–677. ISSN: 0140-9883. DOI: 10.1016/j.eneco.2018.01.003. URL: https://doi.org/10.1016/j.eneco.2018.01.003.

[4] David L McCollum et al. "Energy investment needs for fulfilling the Paris Agreement and achieving the Sustainable Development Goals". In: *Nature Energy* 3.7 (2018), pp. 589–599.

[5] Valentina Bosetti et al. "WITCH - A World Induced Technical Change Hybrid Model". In: *The Energy Journal* 27 (Sept. 2006), pp. 13–37. ISSN: 0195-6574. URL: http://www.jstor.org/stable/23297044 (visited on 09/12/2016).

[6] Johannes Emmerling et al. "The WITCH 2016 model-documentation and implementation of the shared socioeconomic pathways". In: (2016).

[7] Karl E Taylor, Ronald J Stouffer, and Gerald A Meehl. "An overview of CMIP5 and the experiment design". In: *Bulletin of the American meteorological Society* 93.4 (2012), pp. 485–498.

[8] M Rodell et al. "The Global Land Data Assimilation System (GLDAS)". In: *Bulletin of the American Meteorological Society* 85 (2004), pp. 381–394.

[9] John A Hartigan and Manchek A Wong. "AK-means clustering algorithm". In: *Journal of the Royal Statistical Society: Series C (Applied Statistics)* 28.1 (1979), pp. 100–108.

[10] Enrica De Cian and Ian Sue Wing. "Global Energy Consumption in a Warming Climate". In: *Environmental and Resource Economics* 72.2 (2019), pp. 365–410. ISSN: 1573-1502. DOI: 10.1007/s10640-017-0198-4. URL: https://doi.org/10.1007/s10640-017-0198-4.
